# Editorial: Mucosal adaptations to chronic airway injury: mechanisms and interrelationships of epithelial plasticity on innate immunity and airway remodeling

**DOI:** 10.3389/fimmu.2024.1435120

**Published:** 2024-07-09

**Authors:** Allan R. Brasier, Yingxin Zhao, Kian Fan Chung

**Affiliations:** ^1^ Department of Medicine, University of Wisconsin-Madison School of Medicine and Public Health (SMPH), Madison, WI, United States; ^2^ Department of Internal Medicine, University of Texas, Galveston, TX, United States; ^3^ National Heart and Lung Institute, Imperial College London, London, United Kingdom; ^4^ Department of Respiratory Medicine, Royal Brompton and Harefield Hospital, London, United Kingdom

**Keywords:** chronic airway injury, epithelium, repair, innate immunity, epithelial plasticity, airway remodeling

The airway epithelium lying at the external/internal interface and exposed to all the constituents of inhaled air forms a crucial first-line defense against environmental irritants and respiratory pathogens. In response to irritants or pathogens, key sentinel cells in the epithelium trigger innate inflammation, leading to adaptive injury/repair responses. Repeated exposure, leading to cycles of damage, barrier dysfunction and repair are a major, but not fully understood factor in the development and progression of chronic lung diseases such as asthma and chronic obstructive pulmonary disease (COPD). This compendium contains reviews and original research articles that address key cellular and molecular mechanisms behind airway inflammation and remodeling, and the crucial roles these responses play in chronic lung diseases.

The response of the airway to injury is a complex series of interactive pathways which are influenced and often directed by the regionally-specialized epithelial cells present. The cell type composition of the airway epithelium is dependent on regional specialization that collaborate in diverse ways with the cells of the immune system to defend against pathogens and other environmental challenges such as pollutants and to maintain epithelial integrity and stability. In this series of articles, the investigators have addressed different aspects of the epithelial response and adaptation to these external challenges.

Raby et al. have conducted a comprehensive review that provides insights into the significant cellular and structural changes in asthma and COPD. In asthma, epithelial injury and immune activation cause abnormal basal cell differentiation, reduced ciliated cells, goblet cell hyperplasia, and ciliated epithelial cell detachment. This dysregulation contributes to impaired mucociliary clearance and loss of epithelial integrity. In COPD, persistent inflammation and oxidative stress lead to basal cell hyperplasia, goblet cell hyperplasia, and reduced ciliation, contributing to ciliary dysfunction and mucous hyperproduction. In both diseases, a continuous cycle of epithelial injury and immune response activates abnormal tissue repair mechanisms such as epithelial-mesenchymal transition (EMT), fibroblast activation, and airway smooth muscle cell proliferation and hypertrophy, promoting airway remodeling. This review sheds light on the genetic and epigenetic mechanisms underlying airway epithelial dysfunction in these diseases and underscores the importance of developing therapies targeting airway barrier function.

In alignment with this perspective, the study of Chen et al. was focused on the involvement of anoikis-related genes (ANRGs) in the small airway epithelium (SAE) of COPD. It has been well documented that anoikis-resistance is a characteristic of cancer progression and metastasis (1). Because COPD and lung cancer occur as co-morbidities at a higher rate and with the same underlying predispositions (2, 3), it was intriguing to investigate whether anoikis plays a role in the progression of COPD s. The authors investigated the gene expression profiles of SAE of COPD patients and healthy nonsmokers and identified 25 COPD-specific ANRGs. They found that COPD patients could be classified into two subtypes: pro-anoikis and anoikis resistance. Patients with anoikis resistance had more advanced stages of COPD compared to the pro-anoikis group. The study identified Tenomodulin (TNMD) and long intergenic non-protein coding RNA 656 (LINC00656) as important regulators of anoikis resistance in COPD. Furthermore, TNMD was found to have a significant correlation with infiltrating immune cells and abnormal apoptotic signaling pathways, suggesting that targeting ANRGs in airway epithelium could be a potential therapeutic strategy for preventing COPD progression.

Epithelial-mesenchymal plasticity (EMP) is a characteristic feature of chronic lung disease (4). When the epithelium is repeatedly injured, it triggers EMT and the release of fibroblastic growth factors. This disrupts the epithelial barrier function, expands the subepithelial myofibroblast population, and promotes extracellular matrix (ECM) remodeling (4). The study conducted by Brake et al. provides new evidence that EMP plays a critical role in the development of COPD. These researchers found that EMT markers were activated in the small airway epithelium and reticular basement membrane (RBM) of individuals with normal lung function and smoking history (NLFS), current smokers, and ex-smokers with Global Initiative for Chronic Obstructive Lung Disease (GOLD) stage 1 and 2 (COPD-CS and COPD-ES) compared to normal non-smoking controls (NC). This activation was correlated with increased pSMAD2/3 levels and activation of the SMAD pathway in smokers and COPD patients. In contrast, TGF-β1 levels in the small airway tissue of smokers with and without COPD were significantly lower compared to normal non-smoking controls. Although it is well known that TGF-β1 activates the SMAD cassette and subsequentially induces EMT (5), these investigators found that SMAD pathway activation in the small airways of early stage COPD is independent of TGF-β1, indicating that other factors drive EMP at this early stage.

Recent studies have revealed that the chronic activation of the innate immune response (IIR) mediated by NFκB can lead to EMT and airway remodeling in chronic lung disease (4, 6). A review by Pan et al. has highlighted the crucial role of 8-oxoguanine DNA glycosylase 1 (OGG1) in regulating the IIR and EMT through epigenetic mechanisms. OGG1, a DNA repair enzyme, is activated in small airway epithelial cells following injury and is re-purposed to support the DNA binding of transcription factors, such as NFκB, leading to the expression of pro-inflammatory mediators during acute inflammation. With repeated injury and repair, OGG1 facilitates the binding of NFκB, core EMT transcription factors, such as SMADs, and chromatin remodelers, like BRD4 and LSD1, to EMT genes, shifting the inflammatory response towards the expression of pro-fibrotic genes and ECM remodeling enzymes. Therefore, OGG1 is critical in regulating pro-inflammatory and pro-fibrotic responses. The authors discuss recent progress in developing highly selective and specific OGG1 inhibitors for airway remodeling disease, supporting the possibility that this could represent a promising therapeutic target in the clinic.

A review conducted by Kayalar et al. offers comprehensive insights into the effects of particulate matter (PM) of air pollutants on airway injury and epithelial plasticity. Chronic exposure to PM activates the TGF-β-NFκB and Wnt/B-catenin pathways in airway epithelial cells. These pathways activate core EMT transcription regulators such as Twist, Snail, and Zeb, leading to cell-state changes, increased cell mobility, and increased ECM production. The review provides a deeper understanding of the molecular mechanisms involving PM-induced airway inflammation, EMT, and tissue remodeling. It also discusses recent studies on the critical roles of EMP in the pathogenesis of asthma and COPD.

Although much more work needs to be done, these outstanding contributions illustrate the complex cell state dynamics underlying chronic mucosal injury in the airways and provide novel approaches to modifying the outcome of chronic airway disease.

## Author contributions

AB: Writing – original draft, Writing – review & editing. YZ: Writing – original draft, Writing – review & editing. KC: Writing – original draft, Writing – review & editing.
